# Mechanistic study of the effect of potassium ferrate and straw fiber on the enhancement of strength in cement-based solidified municipal sludge

**DOI:** 10.1038/s41598-023-34869-3

**Published:** 2023-05-11

**Authors:** Qiyong Yang, Weixin Xu, Yahong Yang, Xinxia Liu, Qizheng Su, Yangfan Zhang, Ji Wang, Xiang Luo, Mengjing Zhou, Weiping Luo, Haoran Ge

**Affiliations:** 1 Jiangxi Key Laboratory of Industrial Ecological Simulation and Environmental Health in Yangtze River Basin, Jiujiang University, Jiujiang, 332005 Jiangxi China; 2 College of Resources & Environment, Jiujiang University, Jiujiang, 332005 Jiangxi China; 3 School of Civil Engineering, Lanzhou University of Technology, Lanzhou, 730050 Gansu China; 4 Jiujiang Three Gorges, Water Co. LTD, Jiujiang, 332005 Jiangxi China

**Keywords:** Environmental sciences, Engineering, Materials science

## Abstract

The high content of organic matter in sludge is the primary reason for the poor solidifying effect and excessive dosage of the cement base. In this study, potassium ferrate and straw fiber are utilized to synergistically enhance the solidifying effect of the cement and elaborate the strength mechanisms. Among them, potassium ferrate was selected to oxidize and crack the structure of organic matter in sludge and consume part of organic matter; straw fiber was used as an adsorption material to absorb some of the organic material and reduce its interference with the cement hydration reaction; the skeleton function of straw fiber in solidified sludge was used to improve the final solidified sludge strength. It is shown that the presence of these two additives significantly improved the cement solidification strength and reduced the moisture content of the solidified body. Moreover, the moisture content and strength followed an obvious linear relationship (adjusted R^2^ = 0.92), with the strength increasing as the moisture content decreased. After pretreatment with potassium ferrate, the free water content in the dewatered sludge increased by 4.5%, which was conducive to the adequate hydration reaction with cement. The analysis using X-ray diffraction (XRD), scanning electron microscopy with energy dispersive X-ray spectroscopy (SEM/EDS), and mercury intrusion porosimetry (MIP) revealed potassium ferrate synergizes with straw fibers to promote the production of hemihydrate gypsum and gismondine. However, hemihydrate gypsum, calcium carbonate, and gismondine resulted in structural swelling, which was confirmed by the microscopic morphology and pore structure analysis. However, the adverse effects due to swelling were offset by the increase in strength brought by the above crystalline substances.

## Introduction

Sludge contains a high moisture content, high viscosity, and high organic matter content in addition to poor geotechnical properties and toxic and hazardous waste. Presently, 80% of the sludge in China has not yet been stabilized and disposed of safely and properly, posing serious safety hazards and environmental pressure; thus, it is of utmost importance to address the sludge issue.

Sludge solidification as a landfill cover is still a practical disposal method due to its simplicity, economic efficiency, high consumption, and advanced technology. Commonly used binders include ordinary Portland cement, lime, fly ash, slag, and activated magnesium oxide^1–3^. Ordinary Portland cement (OPC) has been recognized as the best curing agent since it is readily available, inexpensive, and simple to incorporate into wet waste^4^. Despite this, there are three major drawbacks while using OPC for sludge solidification. First, cement production can have negative environmental impacts, such as increased greenhouse effect, high energy consumption, and the use of non-renewable resources^5–7^. According to statistics, the cement industry accounts for 6–7% of global CO_2_ emissions^8,9^. Second, the cement-solidified sludge is often high in pH, which is detrimental to groundwater and plant’s growth^10,11^. Last, the hydration reaction of cement is easily disturbed by organic matter in sludge^7,12,13^, significantly reducing the curing effect and requiring additional cement dosages. Thus, a viable strategy involves adding modest amounts of auxiliary additives for regulating cement dosage, minimizing the impact of organic matter on cement hydration reaction, and enhancing the strength of solidified sludge. Currently, auxiliary additives are mainly studied from two perspectives: offsetting or avoiding interference from organic matter with cement hydration and direct oxidation consumption and cracking the structure of organic matter in sludge to reduce interference. In this direction, Zhen et al.^13^ found that the admixture of a small amount of the new aluminate 12CaO·7Al_2_O_3_ crystals counteracted the interference from organic matter and rapidly formed crystals, such as ettringite and calcite with cement hydration products. In another work, Chen et al.^12^ explored that sulfate aluminate cement also directly avoided organic matter interference, generating almost the same amounts of calcium alumina and calcium aluminate gels as the blank control group. However, sulfate aluminate cement is more expensive and is often employed as a silicate cement additive to increase the strength of solidified sludge^14^. In a study by Lei et al.^7^, it was found through the X-ray diffraction measurements that the addition of nano-silica helped to form additional hydrated calcium silicate that counteracted the weakening effect of organic matter on the solidifying strength of the cement. It is clear that there has been an immense amount of research and development on counteracting the hydration reaction of organic matter on cement. Furthermore, Sun^15^ and Li^16^ found that the use of potassium permanganate and potassium persulfate as cementitious additives enhanced the solidifying strength effect, respectively. However, no in-depth study and analysis were conducted in both reports. Therefore, there is still a lot of room for further research in oxidative consumption and cracking the organic matter structure of sludge to enhance the cementitious solidification effect. Potassium ferrate is a special oxidizing agent that is employed in research on sludge dewatering^17^, sludge reduction^18,19^, and wastewater disinfection owing to its powerful oxidizing, flocculating and environmentally friendly properties ^20,21^. Nevertheless, potassium ferrate has rarely been explored for their potentiality in sludge solidification to enhance its strength.

Besides, to further improve the strength and reduce the amount of cement used in solidified sludge, some skeletal materials are often added along with the additives mentioned above to maximize the solidification effect. Commonly used skeleton materials include slag, coal gangue, bentonite, etc.^22–25^; they all contain the active components found in inorganic clay, such as SiO_2_ and Al_2_O_3_. However, all of these compounds must be in a higher alkaline environment to be effective, which will undoubtedly lead to an increase in the cement admixture and a detrimental impact on the environment. In this regard, biomass waste rice straw fiber, with its high yield, low cost, renewable nature, inherent tensile and flexural properties, and high porosity, has the potential to play a role in reinforcing and adsorbing small molecules of organic matter during sludge curing. The high adsorption properties of straw fibers can be fully utilized in conjunction with the strong oxidative properties of potassium ferrate for sludge solidification. Currently, Zhu et al.^26^. have found that straw fibers can improve the effectiveness of cement-based solidifying sludge. On this basis, Yang et al.^27^ have continued to explore the enhancement of the strength of the solidified sludge due to the incorporation of potassium ferrate combined with straw fibers and optimized the suitable ratio of curing agents. However, none of these studies further elaborated on the strength development characteristics and growth mechanism a full explanation of the strength development characteristics and growth mechanism was not provided.

To address the shortcomings of the existing studies, this investigation was devoted to systematically elaborate the strength mechanism of cementitious solidified sludge reinforced synergistically by potassium ferrate in cooperation with straw fiber. Firstly, the macroscopic effects were examined through unconfined compressive strength and moisture content at different curing ages. Following this, the intrinsic mechanism analysis was presented from the perspective of moisture distribution, X-ray diffraction (XRD) patterns, scanning electron microscopy images combined with energy dispersive X-ray spectroscopy (SEM/EDS), and mercury intrusion porosimetry (MIP). Hence, this study offers statistical and theoretical support for applying the proposed process for sludge disposal via sludge solidification.

## Materials and methods

### Characterization of the sludge

The dewatered sludge used in this study was obtained from a domestic wastewater plant in Jiujiang City, Jiangxi Province, China. Table 1 lists its physicochemical properties, while Table 2 displays the findings of the analysis of X-ray fluorescence (XRF) to identify the chemical composition of the sludge.

**Table 1:** Physicochemical properties of the dewatered sludge used in this study.

Property	Moisture content (%)	pH	Organic matter content (wt%)	Heavy metal (g/kg dry sludge)				
				Cr	Cu	Cd	Ni	Zn
Value	74.31–80.15	6.70	45.26	0.102	0.209	0.0005	0.023	0.614

**Table 2:** Chemical compositions of the dewatered sludge used in this study.

Composition	CaO	SiO_2_	Al_2_O_3_	Fe_2_O_3_	SO_3_	MgO	K_2_O	TiO_2_	P_2_O_5_	others
Content (%^a^)	6.39	39.23	23.00	12.86	0.14	0.90	1.96	0.95	11.46	3.11

^a^Dry sludge ash (ignition at 1100 °C).

### Other materials

The binder used in this study was Conch 425 ordinary Portland cement (OPC), and its chemical components are shown in Table 3. The oxidation pretreatment material for sludge was chosen to be potassium ferrate (PF) powder, which is used as a sterilization powder for the common fish pond in the market, having an effective content of 10%. The skeleton material was selected from inexpensive, widely available, and high-yield rice straw fibers (SF) obtained through a 5-mesh sieve screening with an average length of 5 mm.

**Table 3:** Chemical compositions of the ordinary Portland cement.

Composition	CaO	SiO_2_	Al_2_O_3_	Fe_2_O_3_	SO_3_	MgO	K_2_O	TiO_2_	P_2_O_5_	Others
Content (%^a^)	62.65	18.93	4.71	5.47	3.96	1.41	0.59	0.58	0.31	1.39

^a^Ignition at 1100 °C.

### Sample preparation

According to earlier research^27^, 20% OPC, 10% PF, and 5% SF were found to be the appropriate admixtures for each solidification material. Both 20% OPC solidified, and 20% OPC + 10% PF solidified sludge specimens were also prepared for the comparative experiments. The specific mixing ratios and test items are shown in Table 4. The proportion of each solidification material was determined by the weight of the wet sludge, and three solidification samples were collected. The steps for the sample preparation refer to Yang et al.^27^.

**Table 4:** Sample preparation and test program.

Types of solidifiers	Test project	Curing time (days)
20% OPC	UCS	3, 7, 14, 21, 28
	Moisture	3, 7, 14, 21, 28
20% OPC + 10% PF	XRD	28
	SEM/EDS	28
20%OPC + 10%PF + 5%SF	MIP	28

Raw sludge and sludge after oxidation pretreatment should also be tested for moisture distribution.

### Test methods

#### Unconfined compressive strength and moisture content

The unconfined compressive strength was determined from the peak value of the stress–strain curve using the National Standard for Geotechnical testing methods (GB/T 50123-2019). The tests were conducted using a YYW-2 strain-controlled unconfining compression apparatus (Nanjing Ningxi Soil Instruments Co., Ltd.) with a load of 0.6 kN and a loading rate of 2.4 mm/min. The moisture content was set according to the sludge test method for municipal wastewater treatment plants (China National Standard CJ/T 221-2005).

#### Moisture distribution

The sum of interstitial sludge water, hydrated water, and surface adsorbed water was defined as bound water and was determined by centrifugation at 10,000 rpm. In comparison, the free water content was calculated by subtracting the bound water content from the total water content. The main tests were conducted on raw and pretreated sludge with potassium ferrate oxidation.

#### Mineral composition analysis

X-ray diffraction (XRD) was used to investigate the composition of the mineral. Firstly, the samples were soaked in anhydrous ethanol for 24 h to terminate the hydration reaction and then placed in a blast drying oven at 45 °C for 24 h. Lastly, the samples were crushed through a 200-mesh sieve and were then scanned with a Rigaku SmartLab SE X-ray diffractometer with Cu-Kα radiation (λ = 1.54 Å) in the 2θ range of 10° to 80°, a scan speed of 2°/min, a tube voltage of 40 kV, and a current of 40 mA. The XRD patterns were analyzed using MDI Jade 6 software for the phase analysis.

#### Micromorphological and elemental composition analysis

Scanning electron microscopy (SEM) with energy dispersive X-ray spectroscopy (EDS) elemental mapping investigated the microscopic morphology and elemental composition of the samples, respectively. As mentioned for XRD analysis, the hydration reaction of the sample was first terminated with anhydrous ethanol. The samples were then cut into long strips of 5 mm × 5 mm × 20 mm and placed in a freeze dryer at about − 45 °C for 8 h, followed by vacuum drying for 48 h. After drying, the long samples were broken off, and the cross-section of the freshly broken sample was sputter-coated by a thin layer of gold to eliminate the charging effect. Finally, the sample was then examined using a TESCAN MIRA LMS SEM with an accelerating voltage of 3 kV for the morphology and 15 kV for the energy spectrum with an SE2 electron detector.

#### Pore structure analysis

The mercury intrusion porosimetry (MIP) was utilized for the quantitative analysis of pore structure and pore size distribution. After the termination of the hydration reaction, the samples were cut into squares smaller than 15 mm^2^ for freeze-drying treatment. Later, the samples were tested using a MicroActive AutoPore V 9600 capable of finding a pore size in the range of 5 nm‒200 µm and a maximum intrusion pressure of 500 MPa.

## Results and discussion

### Unconfined compressive strength

**Figure 1:**
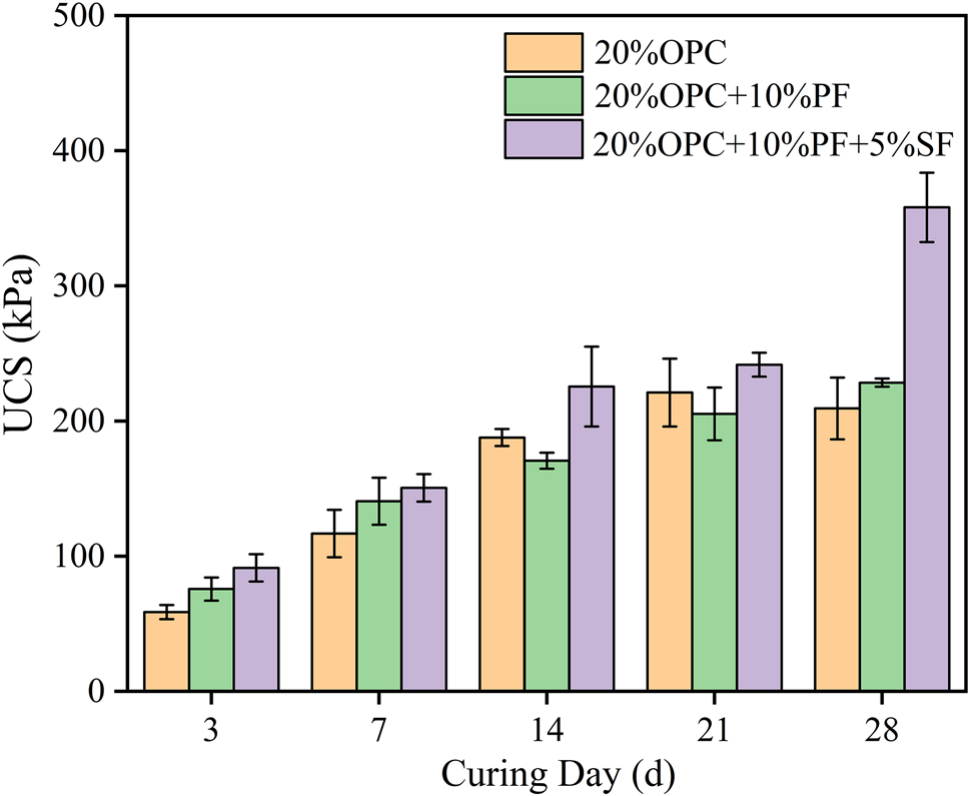
Relationship between curing age and strength.

To compare the effect of solidified sludge with cement and a modified cement-based curing agent, the effects of strength of 20% OPC, 20% OPC + 10% PF, and 20% OPC + 10% PF + 5% SF solidified sludge specimens were studied at different curing ages, and the findings are shown in Fig. 1. It was observed that the strength of 20% OPC was less than the strength of 20% OPC + 10% PF + 5% SF cured sludge at any age, and the difference was the largest after 28 days. While 20% OPC + 10% PF showed a better strength effect than 20% OPC after 3 days, 7 days, and 28 days; however, the strength effect was poorer after 14 days and 21 days, probably due to the instability of the experiment. Despite that, the strength of 20% OPC remained minimal after 28 days indicating the enhancement of the strength effect of cement-based solidified sludge due to the addition of potassium ferrate and straw fiber. The findings revealed that potassium ferrate has strong oxidizing properties, which consumed some organic matter in sludge and promoted cement hydration reaction. Meanwhile, it oxidized organic matter containing sulfur to form inorganic sulfate^28^, which combined with calcium ions produced by cement hydration to form hemihydrate gypsum crystals. In addition, the straw fiber possessed a certain tensile and flexural capacity playing a skeleton-filling role in the solidified sludge. Moreover, the porosity and strong adsorption properties of the straw fiber allowed the adsorption of small molecules of organic matter degraded by the oxidation of potassium ferrate, which further enhanced the hydration effect of cement.

### Moisture content

**Figure 2:**
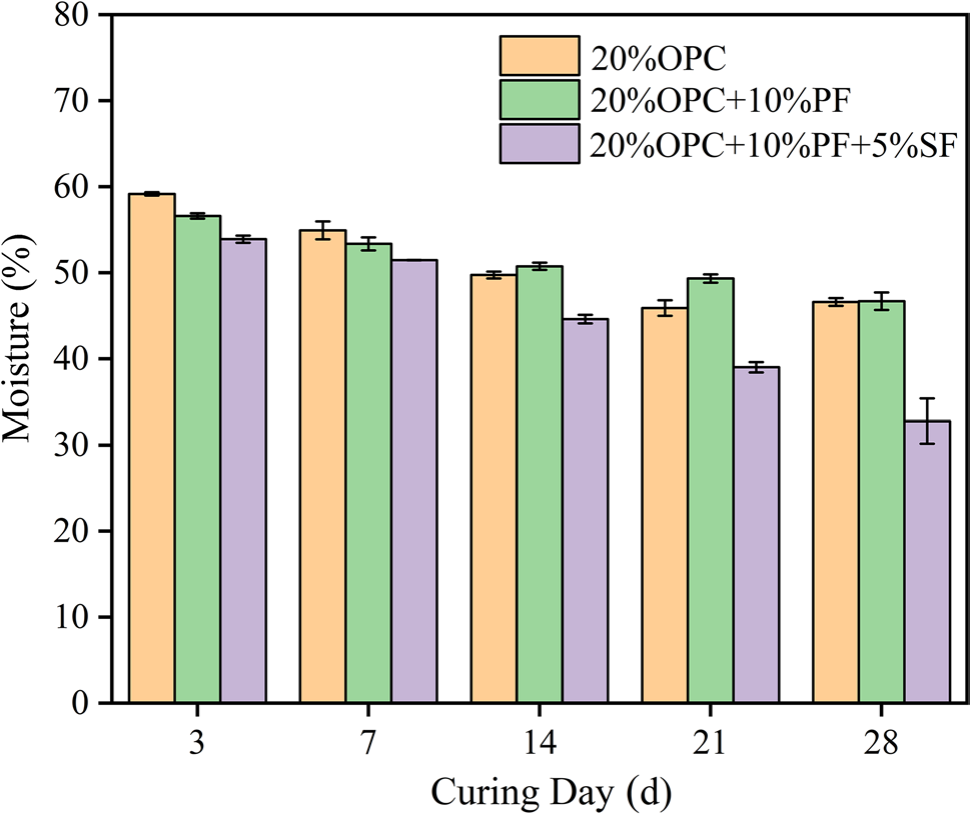
Relationship between curing age and moisture content.

The strength of the solidified sludge is improved by the low moisture content^29^, while the landfill also requires a moisture content of no more than 60%. In this study, the moisture content of the solidified body at different curing ages was investigated, as shown in Fig. 2. The effect of different solidifiers on the reduction of moisture content in sludge and the relationship between moisture content and strength was obtained. It can be seen in Fig. 2 that the moisture content was below 60% to meet the landfill requirements. It was noticed that 20% OPC + 10% PF + 5% SF reduced the moisture content more significantly than 20% OPC at any age, whereas 20% OPC + 10% PF reduced the moisture content, but the effect was not stable and noticeable. This showed that potassium ferrate promoted the reduction of moisture content; however, the straw fiber was required for maximum efficiency. This was attributed to the degradation of organic matter structure in sludge by potassium ferrate releasing a large amount of free water. As free water is easier to evaporate than bound water and has a lower soil‒water potential, thus is more readily consumed by cement and transformed into high-potential energy-bound water^30^, which can effectively reduce cement than 20% OPC. After the conversion of organic matter macromolecules into small molecules by potassium ferrate, the sludge particles overall became more delicate and less permeable, making it difficult to further reduce the water in the sludge. Nevertheless, the addition of straw fiber as a skeleton material greatly increased its porosity and permeability, serving a similar role as a skeleton filter aid in sludge dewatering^31^. At the same time, the straw fiber minimized the sludge water content in combination with the oxidative pretreatment effect of potassium ferrate due to its high porosity.

**Figure 3:**
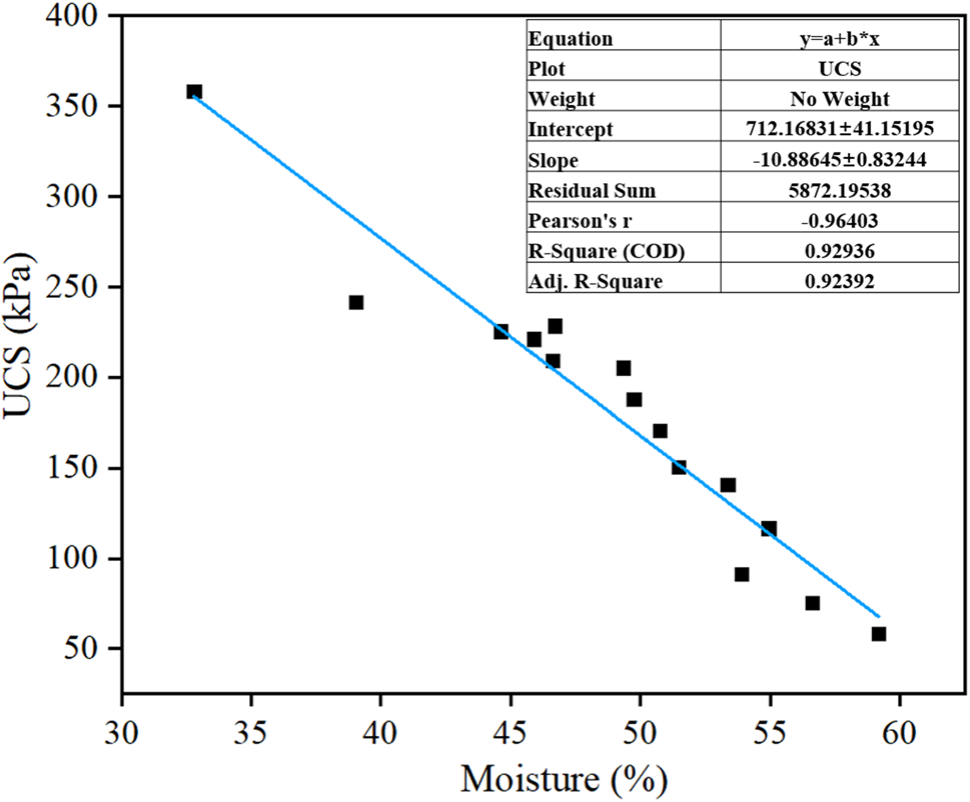
Relationship between the moisture content of solidified body and strength.

The relationship between the moisture content of each solidified body and its unconfined compressive strength is displayed in Fig. 3. It is evident that the moisture content and strength followed an obvious linear relationship (adjusted R^2^ = 0.92), with the strength increasing as the moisture content decreased. This was caused by the fact that when the moisture content dropped, the thickness of the solidified body-bound hydration film also decreased, increasing the inter-particle adsorption force and encouraging sludge agglomeration, which in turn enhanced the strength of the solidified body^29^. This further confirmed that the strength could be effectively improved by lowering the moisture content of the sludge solidification body.

### Moisture distribution

**Figure 4:**
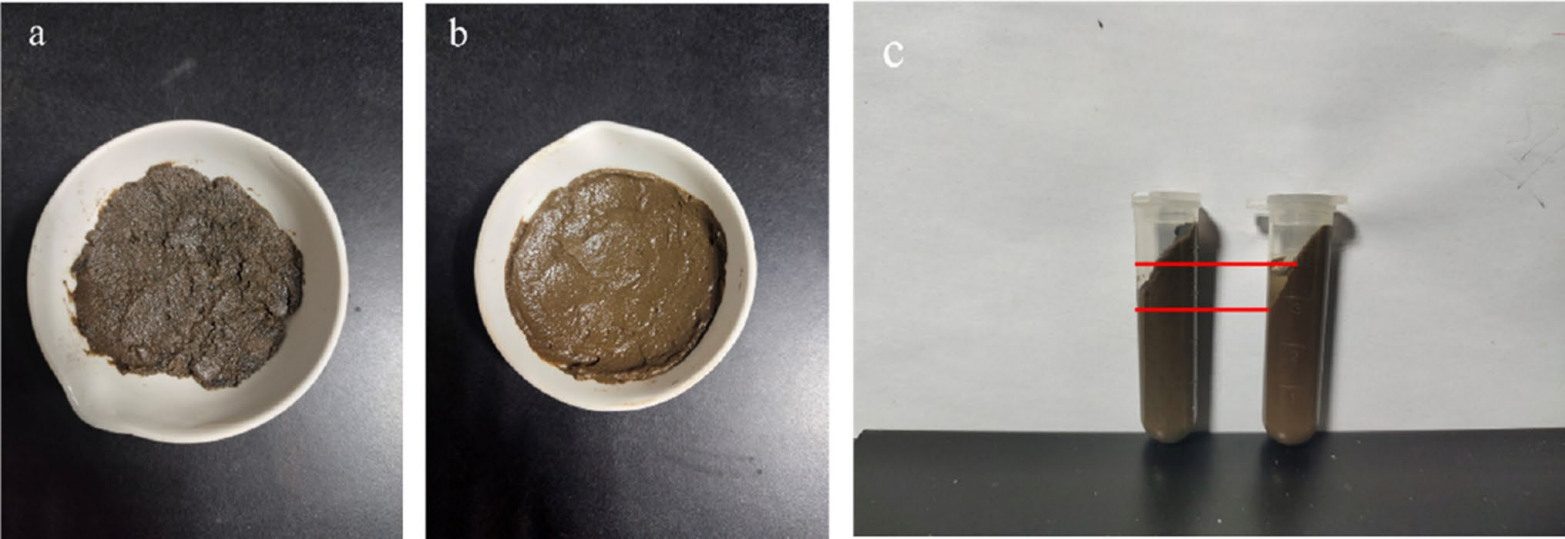
Comparison of raw sludge and oxidized pretreated sludge: (**a**) raw sludge; (**b**) 10% PF pretreatment sludge; (**c**) after centrifugation.

Furthermore, the free water, bound water, and total water content of the raw sludge and pretreated sludge with 10% PF are displayed in Table 5. As can be observed, only 0.01% of the free water was present in the dewatered sludge, with the remaining being bound water. Under ideal conditions, the dewatered sludge does not contain free water^32^, which was consistent with our above-mentioned results. Related studies have revealed that only 3% of the water in sludge was able to hydrate with cement^33^ since the majority of the water is present as bound water. In contrast, after the oxidative pretreatment, the free water content of the sludge increased to 4.95%, while the bound water content decreased to 78.21%. As can be visually seen in Fig. 4a,b that the pretreated sludge is more delicate and moist than the original sludge. Moreover, the solid–liquid stratification and free water content were increased in the pretreated sludge (Fig. 4c), suggesting the degradation of the structure due to potassium ferrate, further allowing the conversion of the bound water to free water. As the free water has low potential energy, which makes it easier to react with cement for hydration reaction, promoting the generation of hydration products^30^. Thus the treatment with potassium ferrate enhanced the effect of solidification strength of the cement. Moreover, the total water content of pretreated sludge decreased slightly, i.e., the initial water content decreased, which was conducive to strength enhancement. The primary cause of the loss of total water content was the increment in the free water content of pretreated sludge, which naturally evaporated during the pretreatment process. Certainly, since the water-cement ratio cannot be too large^34^, the excess free water content was controlled by adding straw fibers. The moisture content was sufficiently reduced during the subsequent maintenance by combining the porous characteristics of the straw fibers and the easy evaporation of free water, which accounts for the lower water content and high strength of 20% OPC + 10% PF + 5% SF compared to the single admixture of 20% OPC.

**Table 5:** Moisture distribution.

Project type	Free water content (%)	Bound water content (%)	Total water content (%)
Raw sludge	0.01	86.13	86.14
10% PF pretreatment sludge	4.95	78.21	83.16

### Minerals analysis

**Figure 5:**
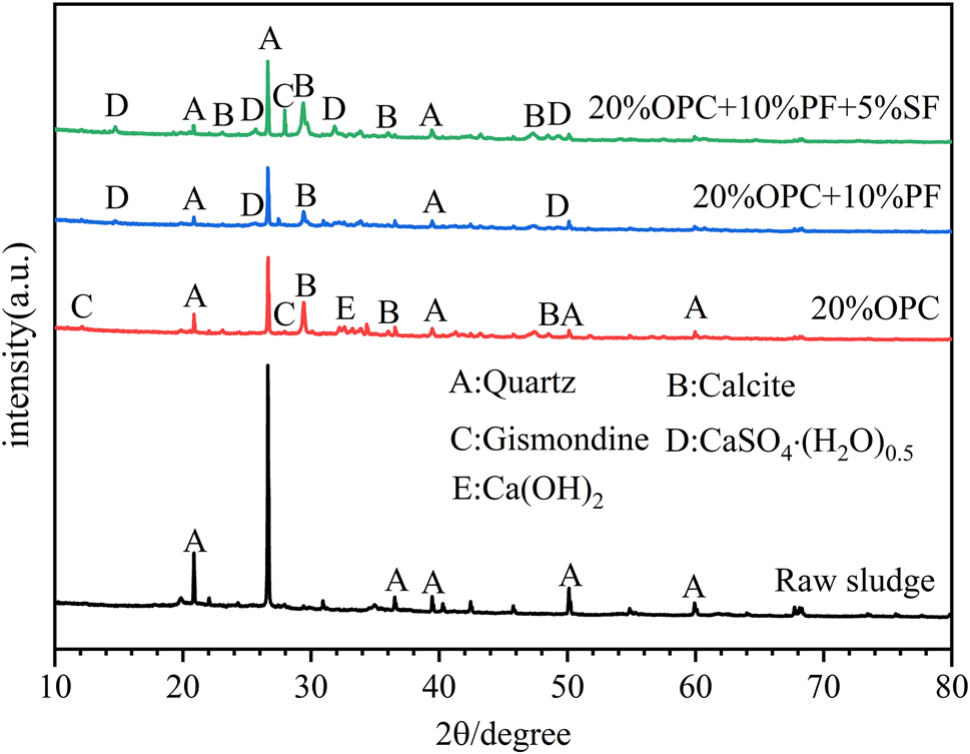
XRD of sludge solidification specimens.

Figure 5 shows the XRD pattern of the raw sludge and 28 days solidified sludge specimen. It was observed that quartz was the main crystal present in the raw sludge. In contrast, the solidified sludge with 20% OPC contained quartz introduced by the sludge, as well as crystals of calcium hydroxide (CH), calcite, and a minimal amount of gismondine crystals due to the hydration reaction. Furthermore, hemihydrate gypsum crystals appeared in the solidified specimen after pretreatment with potassium ferrate, followed by the addition of 20% OPC. In addition a peak was observed at 2θ = 27.45°, which could only be inferred to be calcium-containing crystals in combination with the subsequent elemental composition analysis. Later, when straw fibers were introduced along with potassium ferrate, the XRD peaks of the calcium-containing crystal disappeared; instead, the peak intensity of gismondine crystals dramatically increased. At the same time, the presence of gypsum hemihydrate crystals was also found. Thus, it was concluded that the addition of 20% OPC slightly enhanced the strength of solidified sludge due to the formation of CH, calcite, and gismondine crystals. However, the strength of pretreated sludge with potassium ferrate further improved its strength by generating hemihydrate gypsum and calcium-containing crystals. Besides, due to the high porosity and high adsorption capability of straw fibers, its addition in the sludge with potassium ferrate inhibited the formation of calcium-containing crystals as it was responsible for the adsorption of small organic molecules and some kind of material that forms calcium-containing crystals. This was confirmed by the increase in the peak intensity of the gismondine crystals, thus, enhancing the hydration reaction of cement and improving the overall strength.

### Micromorphological analysis and elemental composition analysis

**Figure 6:**
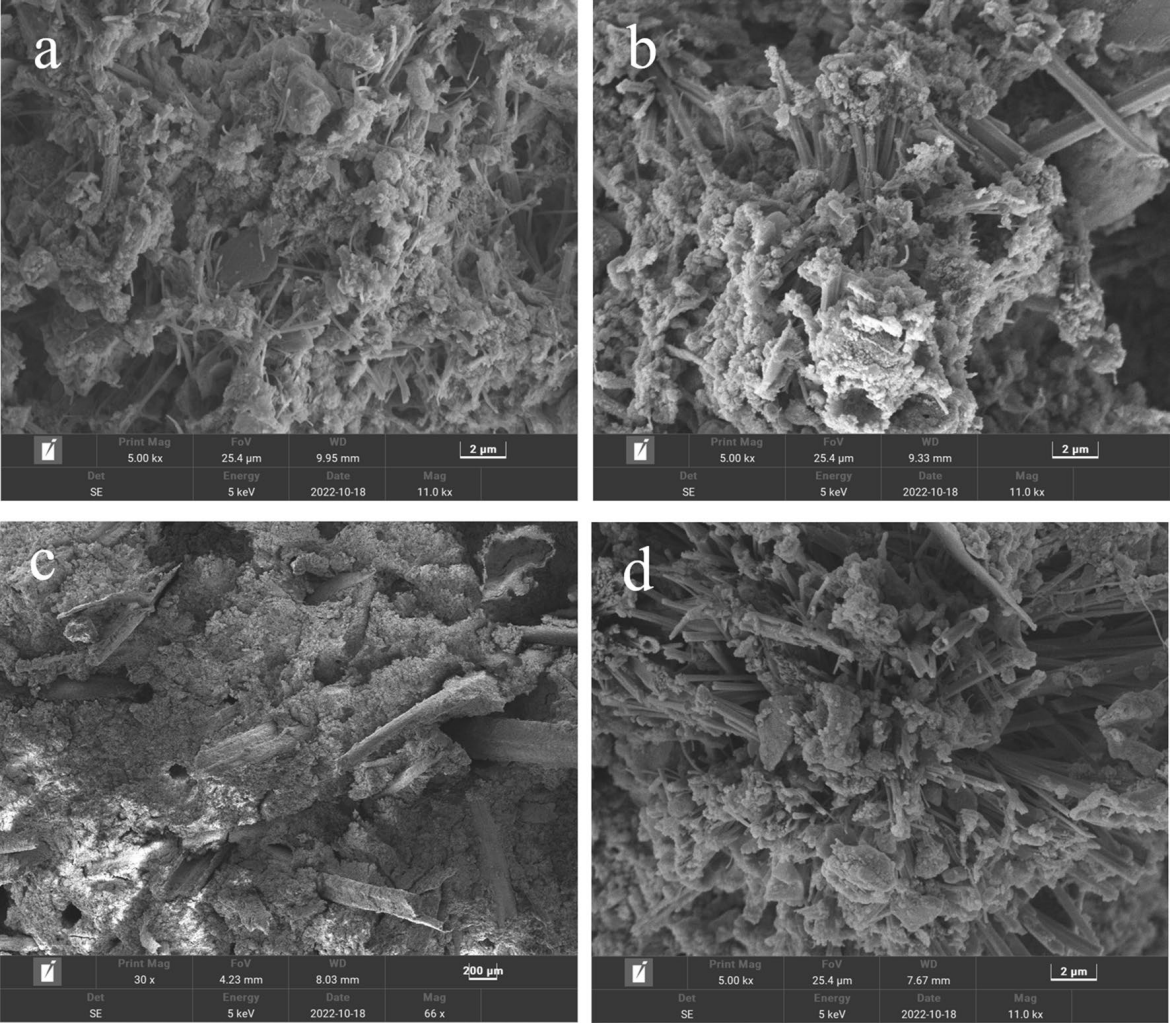
SEM of sludge solidification specimens: (**a**) 20% OPC 5000x; (**b**) 20% OPC + 10% PF 5000x; (**c**) 20% OPC + 10% PF + 5% SF 30x; (**d**) 20% OPC + 10% PF + 5% SF 5000x.

Figure 6 shows the SEM image of the 28 days solidified sludge specimen. As it can be seen that the microstructures of the 20% OPC solidified, sludge are relatively dense, with numerous fibrous gels and massive crystals (Fig. 6a). Acquiring the results from XRD and EDS, it was confirmed that these gels and crystals were hydrated calcium silicate gels, and calcium hydroxide crystals, respectively. These compounds filled the pores and bound the sludge together, strengthening the solidified sludge. In contrast, the microstructures of the solidified sludge containing potassium ferrate and/or straw fibers are more swollen and sparse, as shown in Fig. 6b,d, which to some extent, was not conducive to the development of strength. However, it was evident that both produced long columnar substances, especially in the specimen containing straw fibers. When combined with the macroscopic strength data, it was apparent that these long columnar substances counteracted the negative effects of swelling and thinning. Figure 6c shows the anchoring of the straw fibers in the solidified sludge, where the gelling material holds the sludge together in place, considerably enhancing the strength and toughness of the solidified body. This explained why the late addition of straw fibers had a more significant effect on the strength of cement than the other two.

**Figure 7:**
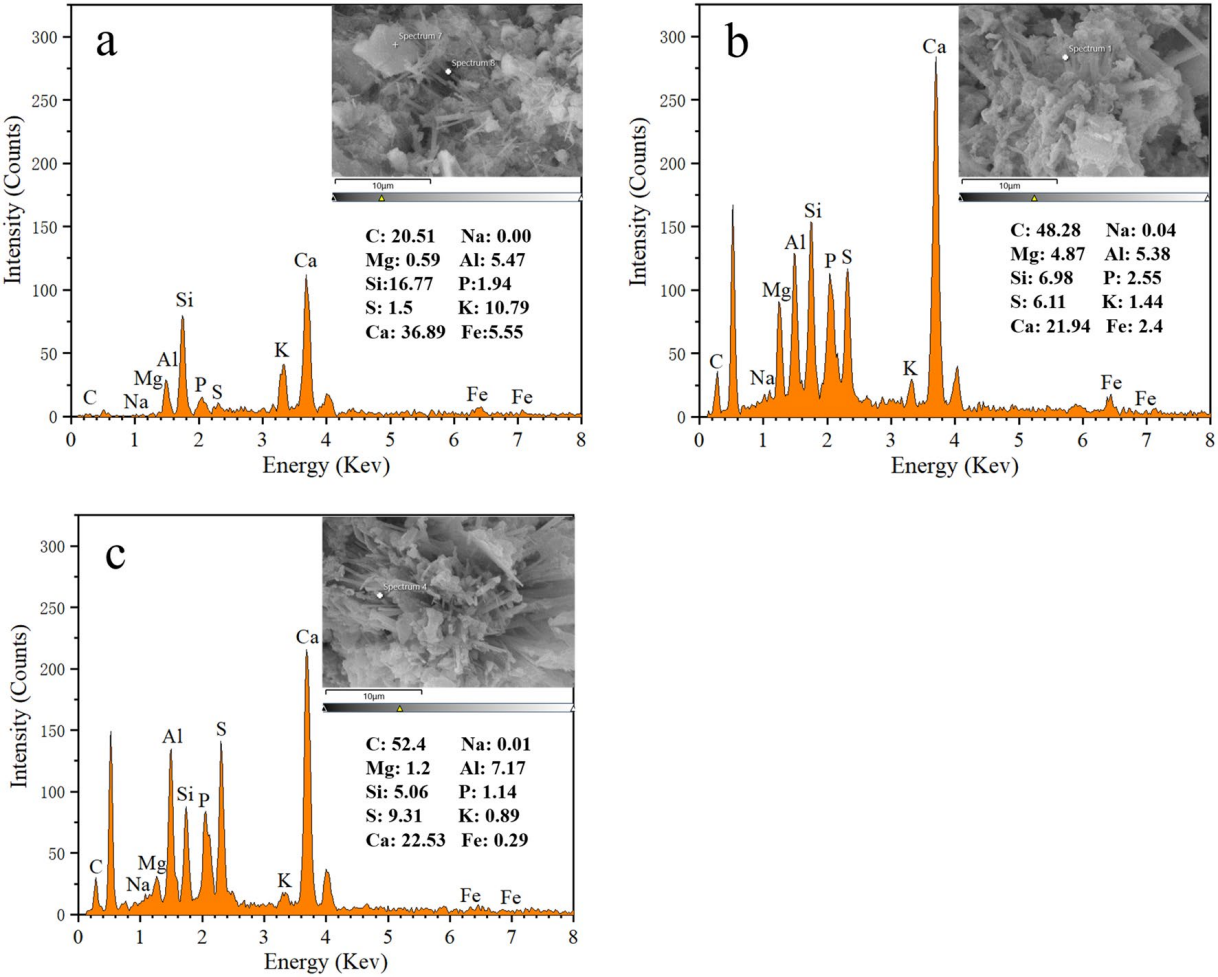
SEM image with its corresponding EDS of solidified sludge: (**a**) 20% OPC; (**b**) 20% OPC + 10% PF; (**c**) 20% OPC + 10% PF + 5% SF.

Next, to identify the elemental composition of the cemented material, the EDS of the three sets of solidified samples were analyzed separately. According to the EDS analysis in Fig. 7a, the main elements of the fibrous cemented material were C, Si, and Ca. As per the earlier studies^5,12^ and the XRD analysis of our results, it was determined that the fibrous gel contained hydrated calcium silicate gel along with some calcium carbonate crystals. Moreover, the composition of the long columnar material in the two sets of solidified specimens was similar, containing C, Al, Si, S, and Ca, as displayed in Fig. 7b,c. Based on the mentioned elements, it was assumed to contain calcium carbonate, gismondine, etc. It was noteworthy that the S content in this sample was 4 to 6 times higher than that of the cement-solidified specimen. Also, when paired with the findings of XRD studies, it was evident that the specimen contained hemihydrate calcium sulfate crystals.

### Pore structure analysis

**Figure 8:**
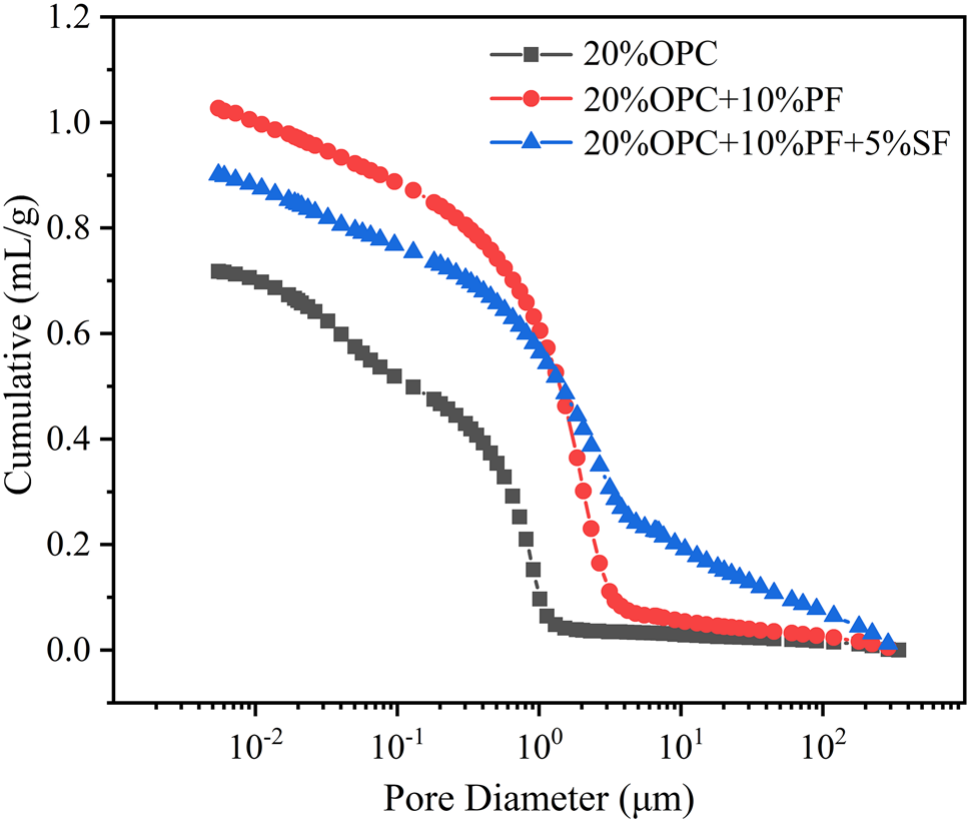
Cumulative pore volume curve.

Furthermore, the cumulative pore volume curves of different solidified sludge specimens after 28 days is shown in Fig. 8. The total pore volume, total porosity, and average pore diameter are quantified in Table 6. The total pore volume of solidified sludge with 20% OPC was the smallest, followed by 20% OPC + 10% PF + 5% SF and 20% OPC + 10% PF in order, which was consistent with the SEM results. It suggested that potassium ferrate and straw fibers did not enhance the strength by reducing its pore volume but rather by forming a significant amount of gismondine, calcium sulfate hemihydrate, and calcium carbonate, as mentioned before. Although this led to volume expansion and increased pore volume, the adverse effects are adequately balanced by the crystal strength. It should be noted here that in areas of resource utilization and construction materials, where high strength (several megapascals or even higher) is needed, the denser solidified body is used^2,12^. Since the strength of the solidified body formed by these compounds drop dramatically if there are too many pores, thus, the adverse effects of crystal expansion cannot be overcome. Consequently, the denser solidified body shows better resultsy^3^.

**Table 6:** Total pore volume, total porosity, and average pore diameter.

Materials	Total pore volume (mL/g)	Total porosity (%)	Average pore diameter (µm)
20% OPC	0.7178	55.63	0.0757
20% OPC + 10% PF	1.0269	64.13	0.1160
20%OPC + 10%PF + 5%PF	0.9013	58.76	0.1110

**Figure 9:**
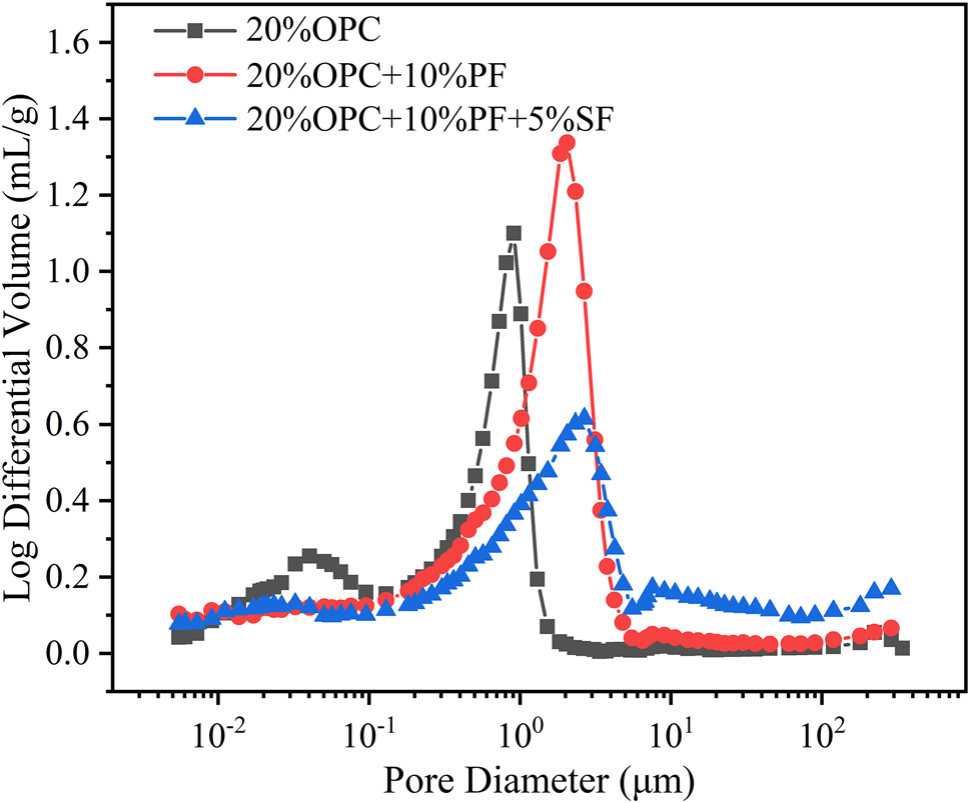
Pore size distribution curve.

**Figure 10:**
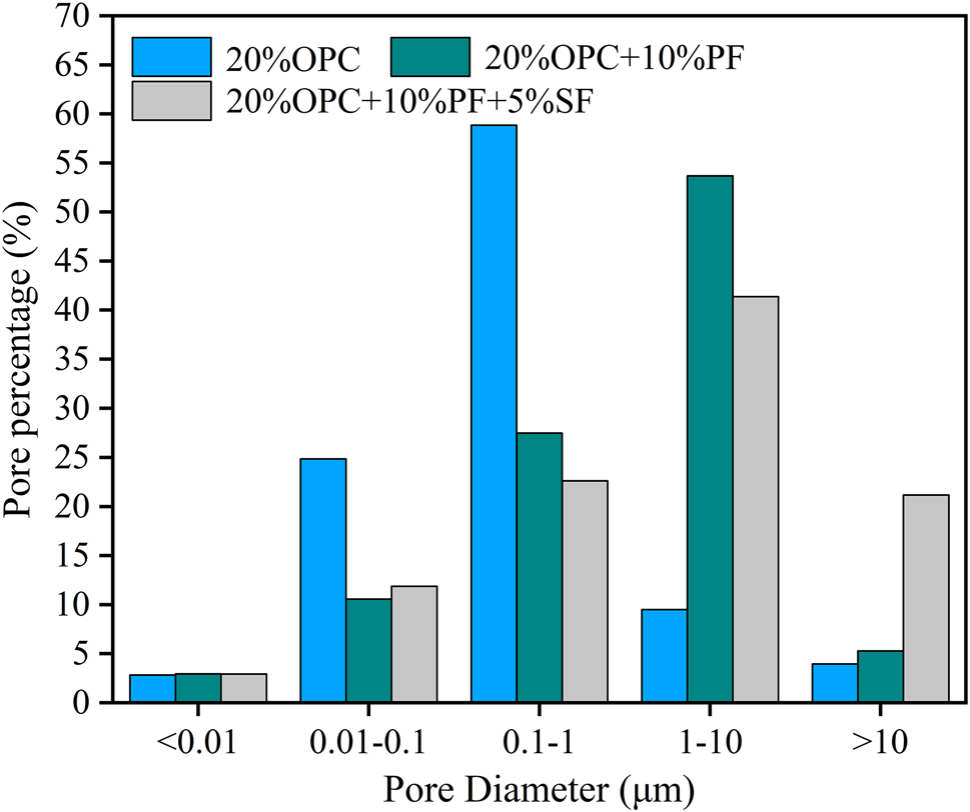
Percentage distribution of pore volume.

Figures 9 and 10 show the pore size distribution curves and pore volume percentages for different solidified sludge after 28 d, respectively. The most probable pore sizes of the sludge specimens solidified with 20% OPC ranged from 0.05 to 1 µm, while the most probable pore sizes of the composite solidified specimens ranged from 1 to 5 µm. Additionally, the pores size was concentrated at 0.01–1 µm when 20% OPC was added. The pore size was concentrated at 1–10 µm when potassium ferrate was used; meanwhile, the percentage of pore size at 0.01–1 µm decreased sharply, with the other changes being negligible. It indicated that the pore size in the range of 0.01–1 µm changed toward 1–10 µm after the addition of potassium ferrate, showing an overall increase in pore volume. As mentioned in the previous section, many long columnar compounds were formed with potassium ferrate, including calcite, calcium sulfate hemihydrate, and/or gismondine, which led to volume expansion. Later, when straw fibers were added, the pore size further increased from 1–10 to > 10 µm increasing the aggregate pore size. Here, the straw fibers, which are themselves porous in nature, served as a skeletal anchoring role in the solidification process, enlarging the aggregate pore size. Therefore, it can be said that both potassium ferrate and straw fibers increased the pore volume during the improvement of the solidification effect, which otherwise has a partial negative impact on the strength. However, the newly generated crystals and the reinforcing and anchoring effect of the straw fibers themselves were sufficient to balance the strength loss and improve the overall strength. Also, it was simpler to reduce the moisture content of the solidified body due to the larger pore size and high permeability, contributing to the strength in another way.

## Conclusion

In summary, the synergistic effect of potassium ferrate (PF) and straw fibers (SF) on the strength of cement-based cured sludge was investigated through a series of macroscopic and microscopic experimental analyses, and the main results of this study are as follows:

1. Compared to 20% OPC solidified sludge, 20% OPC + 10% PF + 5% SF solidified sludge had higher unconfined compressive strength and lower moisture content at any maintenance age condition.
2. Pretreatment of dewatered sludge with potassium ferrate led to an increase in free water content and a decrease in bound water content. The addition of potassium ferrate also facilitated a complete hydration reaction between cement and free water during solidifying. The low potential energy of free water further helped in its faster dissipation during maintenance, reducing the moisture content of the solidified body to enhance strength.
3. Further analysis using XRD and SEM/EDS revealed that compounds such as gismondine, hemihydrate gypsum, calcium carbonate, hydrated calcium silicate, and calcium hydroxide were formed during the treatment of the sludge, which were key components for the strength enhancement of the solidified sludge. It was noted that the potassium ferrate oxidized organic sulfur in the sludge to form hemihydrate gypsum. The small organic molecules were subsequently adsorbed by the straw fibers, promoting the hydration reaction and gismondine formation.
4. The SEM and MIP analysis of morphologies and pore structures showed that the long columnar material composed of gismondine, calcium carbonate, and hemihydrate gypsum supported the structure. It was observed that the composite solidified specimens were more swollen and sparser in morphology and had higher porosity than that of the 20% OPC specimen. However, the total strength of the crystals was sufficient to counteract the effects of swelling.

## Data Availability

All data, models, and code generated or used during the study appear in the submitted article.
